# Myotubularin family phosphatase ceMTM3 is required for muscle maintenance by preventing excessive autophagy in *Caenorhabditis elegans*

**DOI:** 10.1186/1471-2121-13-28

**Published:** 2012-10-31

**Authors:** Xiaokun Yu, Junfeng Ma, Feng Lin, Wanke Zhao, Xueqi Fu, Zhizhuang Joe Zhao

**Affiliations:** 1 Department of Pathology, University of Oklahoma Health Sciences Center, Oklahoma City, Oklahoma, 73104, USA; 2 Edmond H. Fischer Signal Transduction Laboratory, College of Life Sciences, Jilin University, Changchun, 130023, China

**Keywords:** Phosphatase, Myotubalarin, RNAi, Autophagy, Muscle, *C. elegans*, Sarcopenia

## Abstract

**Background:**

Autophagy is a ubiquitous cellular process responsible for the bulk degradation of cytoplasmic components through the autophagosomal-lysosomal pathway. In skeletal muscle, autophagy has been regarded as a key regulator for muscle mass maintenance, and its imbalance leads to sarcopenia. However, the underlying mechanism is poorly understood.

**Results:**

In this study, we demonstrate that ceMTM3, a FYVE-domain containing myotubalarin family phosphatase, is required for the maintenance of muscle fibers by preventing excessive autophagy in *Caenorhabditis elegans*. Knockdown of ceMTM3 by using feeding-based RNA interference caused loss of muscle fibers accompanied by shortening of muscle cell and body size in aged *C. elegans* worms. This was preceded by the occurrence of excessive autophagy in the muscle and other tissues, which subsequently resulted in increased lysosomal activity and necrotic cell death. However, knockdown of ceMTM3 did not aggravate the abnormalities of muscle wasting in autophagy-deficient *atg-18* mutant worms.

**Conclusions:**

Our data suggest an important role of ceMTM3 in regulating autophagy and maintaining muscle fibers. This study may have clinical implications for prevention and treatment of sarcopenia.

## Background

Autophagy is an evolutionarily conserved intracellular process by which cytoplasmic constituents, including long-lived proteins, protein aggregates, organelles, and invading pathogens, are delivered to lysosomes for degradation and subsequent recycling [1]. Autophagy is activated in response to changes in the internal status of the cell and/or changes in the extracellular environment and plays an essential role in cell and tissue homeostasis. Deregulation of autophagy has been linked to many human diseases such as cancer, neurodegeneration, myopathies, diabetes, and infections by bacteria and viruses [2].

Genetic screens, primarily in the yeast *S. cerevisiae*, have identified numerous autophagy-related genes (ATG), many of which have orthologs in higher eukaryotes, including *C. elegans*, *Drosophila*, and mammals [1,3]. The products of these genes constitute two unique protein conjugation systems responsible for formation and elongation of autophagic isolation membranes during autophagy [4]. In addition to the conjugation systems, autophagy initiation is also dependent on phosphatidylinositol 3-phosphate (PI3P) based on the crucial role of the type III PI3Kinase Vps34, an enzyme that generates PI3P and forms a complex with autophagy regulator Beclin-1 (ATG-6) [5]. The level of PI3P is also controlled by PI3P phosphatases that belong to the myotubularin family in mammalian cells [6], which presumably play a role in regulation of autophagy as well. Indeed, studies have demonstrated that PI3P phosphatase Jumpy and myotubularin-related phosphatase 3 (MTMR3) act as negative regulators of autophagy in mammalian cells [7,8].

Myotubularin phosphatases are members of the protein tyrosine phosphatase superfamily [6]. But unlike other protein phosphatases, myotubularin family enzymes dephosphorylate PI3P and phosphatidylinositol (3, 5)-bi-phosphate [6]. Mutations in genes encoding myotubularin proteins are associated with diseases. For example, mutations in MTM1, the founding member of this family, cause X-linked myotubular myopathy (XLMTM), a severe congenital muscular disorder, while mutations in MTMR2 and MTMR13 are associated with Charcot-Marie-Tooth disease [9-11]. We previously have isolated and characterized a *C. elegans* homolog of myotubularin proteins, designated ceMTM3. ceMTM3 preferably dephosphorylates PI3P and contains a FYVE lipid-binding domain at its C-terminus which binds to PI3P [12]. Knockdown of ceMTM3 in *C. elegans* worms by using feeding-based RNA interference caused severe impairment of body movement following post-reproductive age and also significantly shortened their lifespan [12]. We reasoned that this may be related to loss of muscle function due to de-regulation of autophagy. In this study, we demonstrate that knockdown of ceMTM3 induces autophagy that precedes an accelerated loss of muscle fibers in *C. elegans* worms.

## Results and discussion

### Knockdown of ceMTM3 causes loss of muscle fibers in adult *C. elegans*

**Figure 1:**
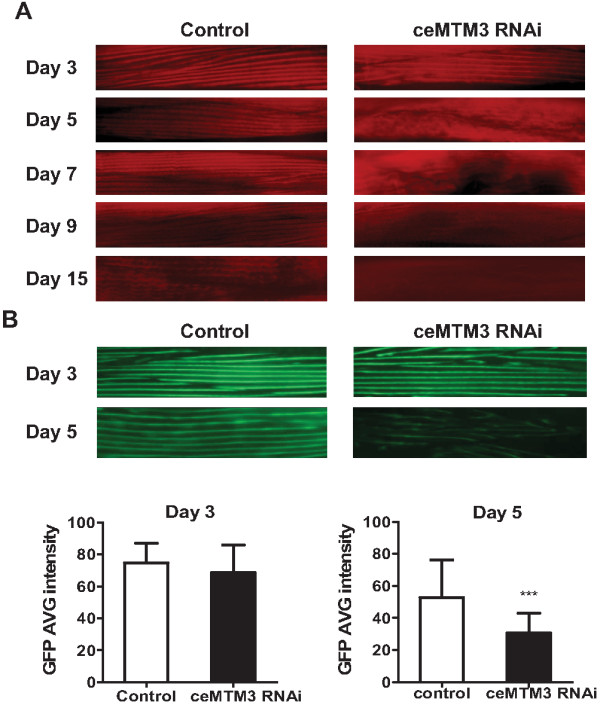
**Knockdown of ceMTM3 causes loss of muscle fibers in adult* C. elegans*.** Normal N2 and RW1596 worms were cultured on NGM plates containing *E. coli* cells carrying vector control or ceMTM3 RNAi from the time they were hatched from eggs. **A**. Alexa Fluor 568- phalloidin staining of muscle fibers in normal N2 worms at indicated ages. **B**. Images of GFP-positive muscle fibers and quantification of GFP intensity in day 3 and day 5 transgenic RW1596 worms which express a GFP::myosin heavy chain A fusion protein. Data represents mean ± SD (n=50). *** p < 0.001.

In an earlier study, we isolated and characterized a *C. elegans* homolog of myotubularin phosphatases, designated ceMTM3 [12]. ceMTM3 is predominately expressed in muscle of adult *C. elegans* worms. It binds PI3P through its C-terminal FYVE domain and preferably dephosphorylates PI3P. Knockdown of ceMTM3 by using feeding-based RNA interference leads to near total loss of ceMTM3 expression and causes a gradual impairment of body movement from day 5 with significant shortening of lifespan of the worms [12]. Since ceMTM3 is predominantly expressed in the muscle [12], the progressive locomotory impairment associated with knockdown of the enzyme may be caused by declining muscle functions. To verify this, we employed Alexa Fluor 568-conjugated phalloidin to detect actin fibers in whole-mount worms (Figure 1A). On day 3, both control and RNAi-treated worms displayed clear and organized actin fibers. However, on day 5, clear deterioration of the fibers was seen with the RNAi-treated worms, and by day 9 the fibers were essentially absent, which correlated with the total impairment of body movement. In contrast, the control worms still maintained actin fiber structure on day 9, although not as organized as that seen with younger worms. By day 15, control worms also displayed significant loss of muscle fibers. Loss of muscle fibers is a progressive event as the worm ages, but knockdown of ceMTM3 markedly accelerates the process. Therefore, our data indicate that ceMTM3 is required to stabilize muscle fibers in adult *C. elegans* worms. To further verify the effects of ceMTM3 knockdown on muscle fibers, we employed RW1596 worms which express GFP:: MHC A carried by a construct in which a GFP coding sequence was inserted at the translation initiation codon in the gene for myosin heavy chain A [13]. The data are shown in Figure 1B. On day 3, both control and RNAi-treated young adult worms showed strong and organized muscle fibers. However, on day 5, while the muscle fibers in control worms displayed a slight decrease, those in the ceMTM RNAi-treated worms were markedly reduced. Quantification of GFP fluorescence signals revealed near 50% loss of GFP-myosin in the treated worms on day 5. The data provide further evidence that knockdown of ceMTM3 destabilize muscle fibers which contain both myosin and actin.

### Knockdown of ceMTM3 shortens the body size of adult *C. elegans*

**Figure 2:**
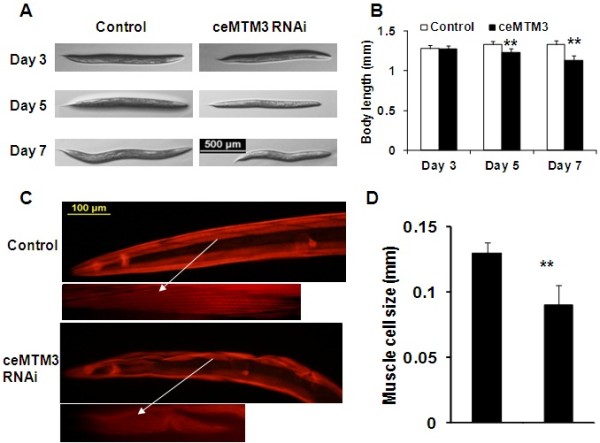
**ceMTM3 knockdown-induced loss of muscle fibers is accompanied by shortening of body size in aged *C. elegans *worms.** Normal N2 worms were cultured on NGM plates containing *E. coli* cells carrying vector control or ceMTM3 RNAi from the time they were hatched from eggs. **A**. Photography of typical worms at day 3, day 5, and day 7. **B**. Statistical comparison of body length of worms. Data represents mean ± SD (n≥12). ** p < 0.01. **C**. Revelation of muscle cells and muscle fibers by Alexa Fluor 568 phalloidin staining of day 5 worms. **D**. Statistical comparison of muscle size of worms. Data represents mean ± SD (n≥14). *** p < 0.001.

Besides the impairment of locomotion, knockdown of ceMTM3 also affected the body size of *C. elegans* worms. As shown in Figure 2A and B, control *C. elegans* entered reproductive period on day 3, but they continued to grow until around day 5 to day 7. However, ceMTM3 RNAi-treated worms started to shrink after day 3. On day 7, the average size of ceMTM3 RNAi-treated worms were 8-15% shorter than the control worms of the same age. The shortening of body length is apparently due to a reduction in the size of individual muscle cells as revealed by staining of muscle actin fibers with Alexa Fluor 568-conjugated phalloidin (Figure 2C). On average, the muscle cell size was reduced by about 30% (Figure 2D). It should be noted that knockdown of ceMTM3 had no significant effects on the development of worms from embryos to young adults (data not shown).

### Knockdown of ceMTM3 causes occurrence of autophagy in multiple cells of adult *C. elegans* worms

**Figure 3:**
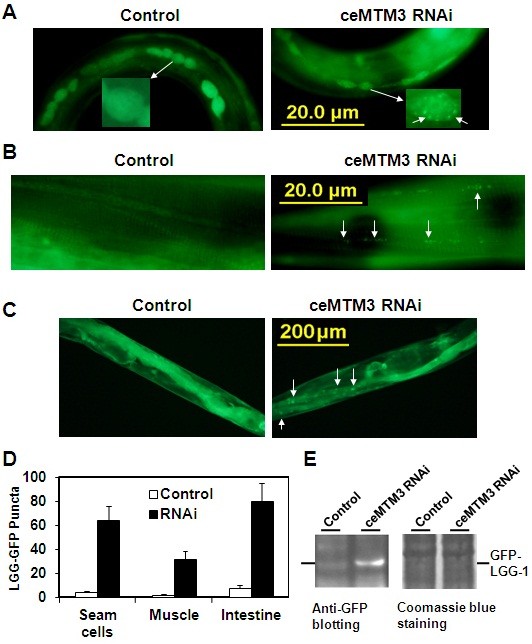
**Knockdown of ceMTM3 causes autophagy in multiples tissues of *C. elegans *worms at different ages.** GFP: LGG-1 worms were cultured on NGM plates containing *E. coli* cells carrying vector control or ceMTM3 RNAi from the time they were hatched from eggs. GFP-positive puncta (indicated by arrows) represent autophagosomes which are abundant in ceMTM3 RNAi-treated worms. **A**. Autophagosomes in seam cells of day 2 worm larvae. **B**. Autophagosomes in muscle cells of day 3 young adult worms. Note that muscle fiber bands are visible in the background. **C**. Autophagosomes in intestine on day 5 adult worms. **D**. Average numbers of GFP puncta in 4 seam cells, 60 micrometer-long muscles, and entire intestine. Error bars denote standard deviation, n≥20, p<0.001). **E**. Western blotting analyses of lipid-bound GFP-LGG-1. Control and ceMTM3 RNAi-treated worms (mixed ages) were extracted in phosphate-buffer saline by sonication. After centrifugation at 800 g for 10 min, the supernatants were collected and subjected to further centrifugation at 100,000g for 1 h. The pellets were extracted in SDS gel sample buffer and directly used for western blotting analysis with an anti-GFP antibody. The position of GFP-LGG-1 is indicated. Equal protein loading is demonstrated by Coomassie blue staining.

We hypothesized that the loss of muscle fibers may be caused by death of muscle cells due to autophagy, which is known to have an important role in muscle maintenance and to be dependent on PI3P [5,14]. Our earlier studies have shown the knockdown of ceMTM3 affects the total level of PI3P in *C. elegans* worms [12]. It is likely that loss of ceMTM function causes autophagy, thereby leading to loss of muscle fibers. To visualize autophagy, we used worms carrying a GFP::LGG-1 fusion protein. LGG-1 is an ortholog of yeast Atg8p and mammalian MAP-LC3 in *C. elegans*. During autophagy, GFP-labeled LGG-1 protein localizes to preautophagosomal and autophagosomal membranes and forms green puncta, thereby serving as a reporter for autophagy [15]. Data shown in Figure 3 demonstrate occurrence of autophagy in multiple cells in ceMTM3 RNAi-treated worms on different days. Autophagy occurred in seam cells in day 2 larvae, in muscle cells in day 3 young adults, and day 4 and thereafter in intestine of adult worms (Figure 3A-D). In the control worm, such structures were essentially absent until day 12 when a few sporadic punctuates appeared in the intestine (not shown). We also confirmed induction of autophagy by detecting lipidation of GFP-LGG-1, another hallmark of autophagy. As shown in Figure 3E, upon treatment of *C. elegans* worms with ceMTM3 RNAi, a clear band of ~37 kDa was recognized by anti-GFP antibody in the membrane extracts of worms. Together, our data indicate that knockdown of ceMTM3 caused a marked increase in autophagy activity. It is worth noting that the onset of autophagy apparently preceded the deterioration of muscle fibers which began on day 5 (see Figure 1). Apparently, the presence of ceMTM3 prevents occurrence of autophagy in the normal worms as they age. The occurrence of autophagy in muscle and intestine is consistent with the distributions of the enzyme in these tissues as revealed in our earlier studies [12].

### Knockdown ceMTM3 increases lysosomal activities and induces necrotic cell death in *C. elegans*

**Figure 4:**
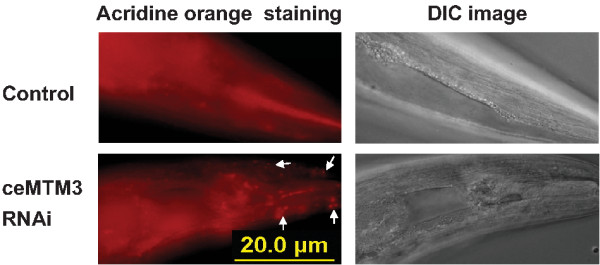
**Knockdown of ceMTM3 induces increased lysosomal activity.** Day 5 adult worms were treated with acridine orange on NGM plates. Data represent fluorescence and DIC images of the same worm sections. Arrows indicate lysosomes which are abundant in ceMTM3 RNAi-treated worms. Note that muscle fibers are visible in DIC images, indicating increased numbers of lysosomes are present in muscle cells.

**Figure 5:**
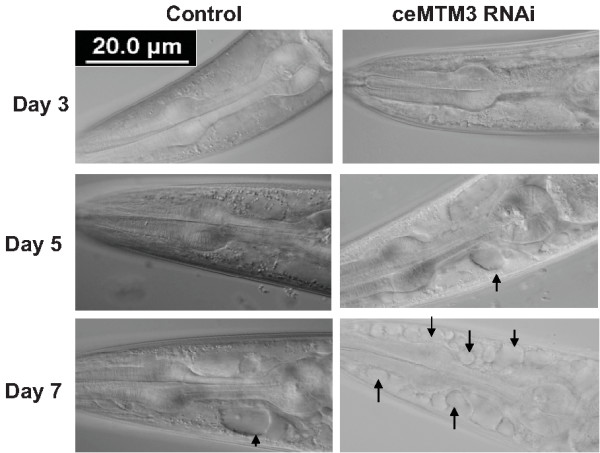
**Knockdown of ceMTM3 causes necrotic cell death in aged *C. elegans.*** DIC images of the head regions of control and cMTM3 RNAi-treated worms at indicated ages. Arrows point to the positions of necrotic cells.

After formation, autophagosomes deliver the sequestered cytoplasmic materials into lysosomes for further degradation [1]. To find out whether the formation of autophagosomes induced by knockdown of ceMTM3 has further functional consequences, we examined the lysosomal activities by using acridine orange, which is commonly used for staining of lysosomes [16,17]. The results showed that knockdown of ceMTM3 induced appearance of numerous lysosomal vesicles in the head region of day 4 adult worms (Figure 4). These vesicles were apparently in muscle cells as the muscle fibers were visible in DIC images. The lysosomal vesicles were also seen in the body wall muscle although less predominant (not shown). Excessive lysosomes induce cytoplasmic acidification and further lead to necrotic cell death. In fact, studies have demonstrated that autophagy is required for necrotic cell death in *C. elegans*[18]. As expected, knockdown of ceMTM3 caused significant necrosis as shown in Figure 5. In the head region of ceMTM3 RNAi-treated worms, necrotic cell death, as indicated by cell corpses, started to appear on day 5 and became overwhelming by day 7. However, this was not seen in control worms, although sporadic cell necrotic cell death was also occasionally observed on day 7 and thereafter. On average, control day 7 worms displayed 0.3±0.2 corpses in the head region while ceMTM3 RNAi-treated worms of the same age showed 4±0.6 corpses (p<0.001). Necrotic cell death was also seen in the body wall muscle of RNAi-treated worms on day 7 (not shown).

### Knockdown ceMTM3 does not aggravate the abnormalities of muscle wasting in autophagy-deficient worms

**Figure 6:**
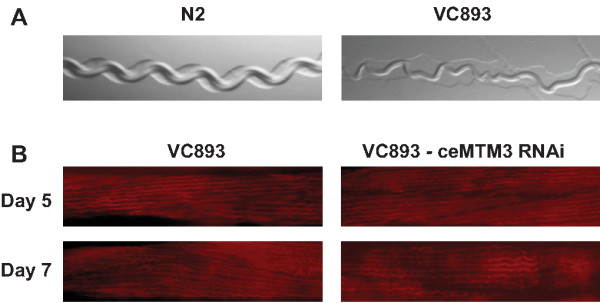
**Knockdown of ceMTM3 does not aggravate the abnormalities of muscle wasting in *atg-18 *mutant worms.** VC893 *atg-18* mutant worms were cultured on NGM plates containing *E. coli* cells carrying vector control or ceMTM3 RNAi from the time they were hatched from eggs. **A**. Moving tracks of day 5 N2 and VC893 worms cultured under the normal condition. **B**. Alexa Fluor 568 phalloidin staining of muscle fibers in control and ceMTM3 RNAi-treated worms of indicated ages. In comparison with normal N2 worms shown in Figure 1, VC893 worms displayed dimmer and less organized muscle fiber staining.

The above data suggest knockdown of ceMTM3 function leads to autophagy which in turn causes cell death and loss of muscle fibers. To further address the causal relationship between loss of muscle fibers and onset of excessive autophagy, we employed autophagy deficient VC893 worms. These worms lack ATP-18, an ortholog of yeast Atg18p and human WIPI, and thus are not able to recruit autophagic membranes to form autophagosomes [18,19]. Interestingly, VC893 worms fed on normal *E. coli* food exhibited impaired locomotion after day 5 (Figure 6A). This was associated with deterioration of muscle fibers as revealed by Alexa Fluor 568-conjugated phalloidin staining (Figure 6B, see also Figure 1A for comparison). Nonetheless, knockdown of ceMTM3 did not further exacerbate this abnormality, implying that induction of autophagy is required for ceMTM3 RNAi-induced muscle fiber loss.

The data described above demonstrated that knockdown of a MTM family enzyme causes autophagy and subsequently causes loss of muscle fibers. As a PI3P phosphatase, ceMTM3 should play an important role in controlling the level of PI3P. The involvement of PI3P in autophagy has been demonstrated for the most part by studying the type III PI3Kinase Vps34. Vps34 is a type III PI3 kinase responsible for generation of PI3P. It forms a complex with its autophagy-regulatory partner Beclin-1 (ATG-6), thereby initiating autophagy [5]. Vps34 also has an essential role in membrane trafficking and endocytosis, and Vps34 mutant worms display lethality and molting defects as well as alterations in the outer nuclear membrane and in the endoplasmic reticulum [20,21]. Our current study demonstrated that knockdown of ceMTM3, a PI3P phosphatase predominately expressed in the muscle, results in autophagy which subsequently leads to loss of muscle fibers. In a sense, one may postulate that inactivation of ceMTM3 is equivalent to activation of VPS34 in regulating the autophagy process. Therefore, PI3P phosphatases and PI3 kinases play equally important roles in regulation of autophagy.

The role of autophagy in muscle wasting and maintenance has been well documented [14]. Autophagy appears to be a double edged sword. Too much or too little of it can cause muscle weakness and atrophy. For example, excessive autophagy, induced by denervation and fasting, contributes to muscle atrophy [22]. By demonstrating that occurrence of autophagy upon knockdown of ceMTM3 precedes muscle fiber loss, our study provides further proof of the harmful effects of too much autophagy in muscle function. On the other hand, deficiency in autophagy is also known to cause muscle atrophy. For example, both Atg5^−/−^ and Atg7^−/−^ mice show muscle loss accompanied by accumulation of protein aggregates and abnormal membranous structures in the muscle cells [23,24]. By the same concept, our study demonstrated that autophagy-deficient *atg-18* worms exhibited impaired body movement and loss of muscle fibers (Figure 6). In addition, an earlier study demonstrated that loss of autophagy function due to inactivation of unc-51/atg1 and bec-1/atg6/beclin1 results in small body size without affecting cell number [25]. Our current data demonstrated that excessive autophagy associated with knockdown of ceMTM3 also causes shortening of body size with smaller muscle cells. Therefore, autophagy appears to be a dynamic process and should be kept in a balance. From a therapeutic perspective, it is important to understand whether the reduction of autophagy is helpful during muscle loss or whether enhancement of autophagosome flux is beneficial for the clearance of dangerous organelles or toxic proteins. To keep healthy muscle function, we need to keep a normal level of autophagy flux to rejuvenate organelles and to remove dysfunctional mitochondria and ER membranes but to avoid excessive autophagy which may lead to breakdown of normal muscle fibers.

The loss of muscle mass, referred to as sarcopenia, is a normal phenomenon in animals as a consequence of aging [26]. For humans, decrease in muscle tissue begins around the age of 50 years and becomes more dramatic beyond the 60th year of life. For *C. elegans* worms, loss of muscle fibers and consequent impairment of body movement is clearly seen after day 15 and is much accelerated by knockdown of ceMTM3 (Figure 1). Aging is associated with a progressive decline of muscle mass, strength, and quality, but the mechanism underlying the muscle wasting remains unresolved [27]. Studies have shown that sarcopenia is not due to lack of regenerative drive in senescent skeletal muscle [28], implying that the maintenance of existing muscle is crucial. Among the many factors correlating with sarcopenia during aging, oxidative stress has been extensively investigated [29]. We believe that oxidation stress-induced inactivation of MTM family phosphatases may play a major role in the muscle wasting process. Like all the other members of the tyrosine phosphatase superfamily, MTM family enzymes are susceptible to oxidation-induced inactivation because they contain a highly reactive cysteinyl residue at the catalytic center. Reactive oxygen species, such as the superoxide radical (O_2_^-·^), hydrogen peroxide (H_2_O_2_), the hydroxyl radical (OH^·^), and nitric oxide (NO) are produced in muscle at rest, and this generation is increased by contractile activity [30]. Oxidative damage is considered the main cause of aging. In fact, it was thought that a slower accumulation of oxidative damage is at least partly responsible for the life extension effects seen in *C. elegans* with *daf-2* and *age-1* mutations [31]. Our earlier data have shown that ceMTM3 is sensitive to peroxide and loss of ceMTM3 function causes muscle deterioration in *C. elegans*[12]. Therefore, inactivation of ceMTM3 by oxidation may be attributable to loss of muscle fibers in normal worms at later ages. Thus, to prevent sarcopenia, it is important to maintain a normal level of PI3P phosphatase activity.

## Conclusion

By using the C. elegans worm as a model system, our study provides further evidence that the MTM family phosphatases play a negative role in autophagy. Our study also demonstrates a correlation between excessive autophagy and muscle wasting, implying a normal level of autophagy is important for muscle function and maintenance. Considering sensitivity of tyrosine phosphatase superfamily enzymes to reactive oxygen species, loss of muscle fibers during normal aging may be a consequence of PI3P phosphatase inactivation. Therefore, studying the MTM enzymes may have clinical implications in the prevention and treatment of human sarcopenia.

## Methods

### Nematode strains and maintenance

*C. elegans* worms were grown on nematode growth medium (NGM) plates with abundant *E. coli* food at 20°C according to standard protocols. Wild-type Bristol N2, ATG-18-deficient strain VC893 *atg-18*(gk378) V], and GFP::myosin heavy chain A-expressing RW1596 [myo-3(*st386*) V; stEx30] worms were obtained from the Caenorhabditis Genetics Center. Transgenic N2 worms [Plgg-1::gfp::lgg-1+rol-6] carrying the fusion protein GFP::LGG-1 autophagy marker were kindly provided by Dr. Beth Levine (University of Texas Southwestern Medical School) [15]. All worms were scored at the same chronological age and were moved to new plates every day after they reached the reproductive period to avoid progeny contamination. Day 0 refers to the laid egg stage.

### Knockdown of ceMTM3

The full-length coding sequence of ceMTM3 (−6 to 2,910 with translation starting codon ATG starting from 1) was cloned into the pPD129.36 vector. Plain p129.36 vector was used as control throughout the study. The HT115 (DE3) *E. coli* cells were employed as hosts for expression of double-stranded RNAs, and 0.4 mM IPTG was used to induce expression of dsRNA. The efficiency of RNAi-medicated knockdown was confirmed by western blotting with anti-ceMTM3 antibodies as previously described [12].

### Staining of worms and microscopy

For phalloidin staining of actin filaments, worms were fixed with cold acetone and then stained with Alexa Fluor 568-phalloidin. For acridine orange staining, 500 ul of 0.01 mg/ml acridine orange made in M9 buffer was added directly onto worms cultured in a 6 cm NGM plate to ensure the entire plates were evenly covered. After incubation at 20°C in the dark for 1 h, the worms were transferred to a new culture plate without acridine orange and allowed to recover at 20°C for 1 h before being immobilized with sodium azide for microscopic analysis [16]. For visualization of fluorescence in GFP-positive worms, worms were treated with sodium azide and then viewed under a fluorescent microscope with a GFP filter. All microscopic analysis was performed with an Olympus BX51 microscope equipped with DIC lens. Digital images were captured using an Olympus DP71 camera with the DP-BSW application software (version 03.02). Quantification of fluorescent signals was performed by using the FluorChem SP program from Alpha Innotech.

### Statistical analysis

Statistical comparison between control and treatment groups was performed with unpaired *t* test using the GraphPad Prism software (Graphpad Software, La Jolla, CA). Differences with p<0.05 were defined significant.

## Abbreviations

GFP: Green fluorescent protein; NGM: Nematode growth medium; PI3P: Phosphatidylinositol 3-phosphate; RNAi: RNA interference.

## Competing interests

The authors declare no conflict of interests.

## Authors’ contributions

XY, JM, FL, and WZ performed the research; XF designed the research; ZJZ designed and supervised the research. All authors wrote, read, and approved the manuscript.
